# Supplementary material for: The interactome of intact mitochondria by cross-linking mass spectrometry provides evidence for coexisting respiratory supercomplexes
Source: Mol Cell Proteomics. 2017 Dec 8;17(2):216–32. doi: 10.1074/mcp.RA117.000470 (PMC5795388; doi:10.1074/mcp.RA117.000470)
Supplement: Supplemental Data [file supp_RA117.000470_133922_0_supp_23978_fzffwf.zip › spectra_annotation/mito_DR_spectra_annotation/259-1-1-1-9-1.pdf]

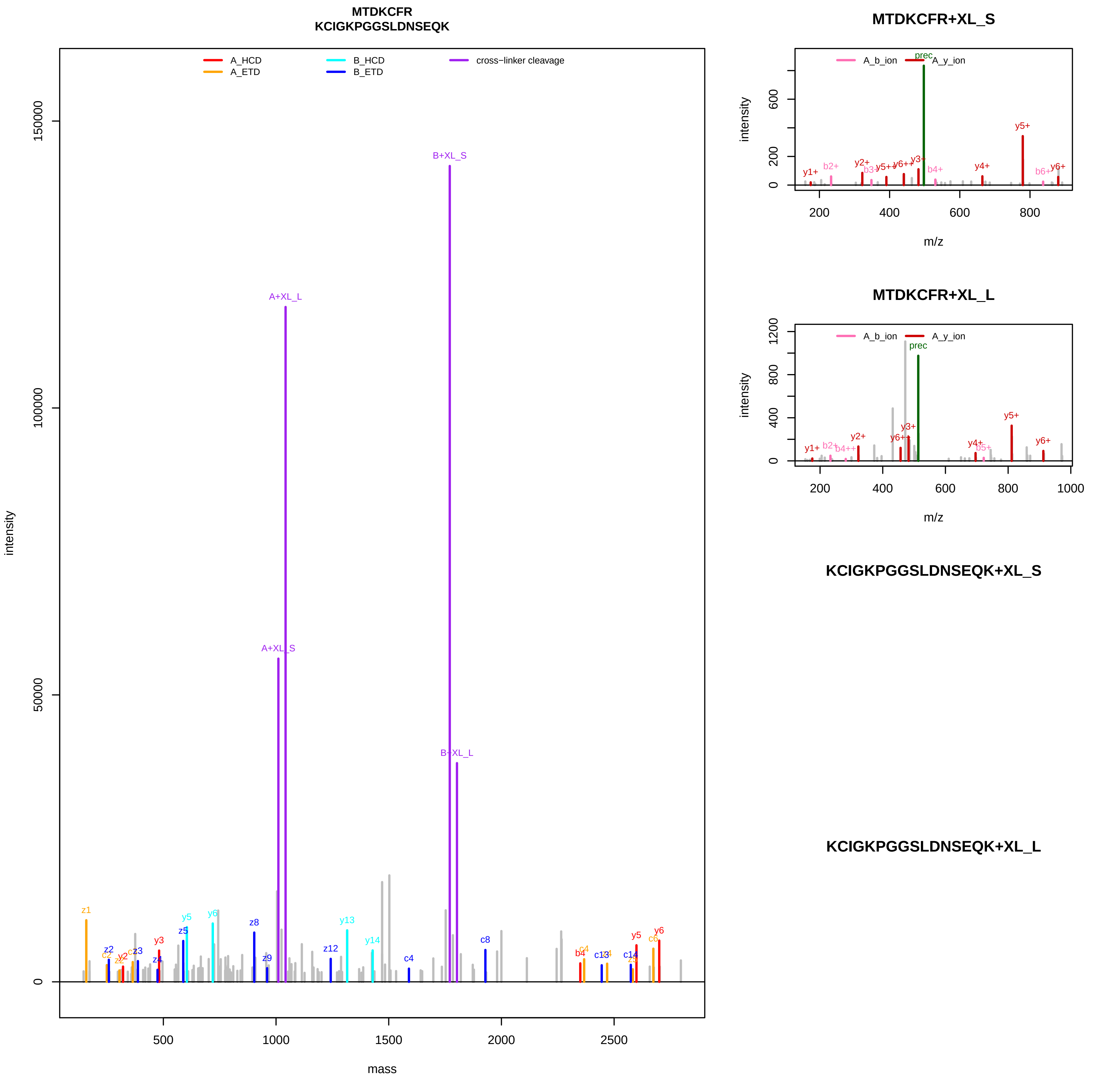
MTDKCFR
KCIGKPGGSLDNSEQK
A_HCD
A_ETD
B_HCD
B_ETD
cross−linker cleavage
B+XL_S
A+XL_L
A+XL_S
B+XL_L
intensity
mass
150000
100000
50000
0
500
1000
1500
2000
2500
z1
c2
z2
y2
z3
y3
z4
z5
y5
y6
z8
z9
z12
y13
y14
c4
c8
b4
c13
c14
y5
c6
y6
MTDKCFR+XL_S
A_b_ion
A_y_ion
prec
y1+
b2+
y2+
b3+
y5++
y6++
y3+
b4+
y4+
y5+
b6+
y6+
intensity
m/z
0
200
600
200
400
600
800
MTDKCFR+XL_L
A_b_ion
A_y_ion
prec
y1+
b2+
b4++
y2+
y6++
y3+
y4+
b5+
y5+
y6+
intensity
m/z
0
400
800
1200
200
400
600
800
1000
KCIGKPGGSLDNSEQK+XL_S
KCIGKPGGSLDNSEQK+XL_L
